# Psychophysics of a Nociceptive Test in the Mouse: Ambient Temperature as a Key Factor for Variation

**DOI:** 10.1371/journal.pone.0036699

**Published:** 2012-05-18

**Authors:** Ivanne Pincedé, Bernard Pollin, Theo Meert, Léon Plaghki, Daniel Le Bars

**Affiliations:** 1 Team “Pain", INSERM UMRS 975, CNRS UMR 7225, Paris, France; 2 Université Pierre et Marie Curie, Faculté de Médecine UPMC, Paris, France; 3 Department of Psychology, University of Leuven, Leuven, Belgium; 4 Unité READ, Université Catholique de Louvain, Brussels, Belgium; King’s College London, United Kingdom

## Abstract

**Background:**

The mouse is increasingly used in biomedical research, notably in behavioral neurosciences for the development of tests or models of pain. Our goal was to provide the scientific community with an outstanding tool that allows the determination of psychophysical descriptors of a nociceptive reaction, which are inaccessible with conventional methods: namely the true threshold, true latency, conduction velocity of the peripheral fibers that trigger the response and latency of the central decision-making process.

**Methodology/Principal Findings:**

Basically, the procedures involved heating of the tail with a CO_2_ laser, recording of tail temperature with an infrared camera and stopping the heating when the animal reacted. The method is based mainly on the measurement of three observable variables, namely the initial temperature, the heating rate and the temperature reached at the actual moment of the reaction following random variations in noxious radiant heat. The initial temperature of the tail, which itself depends on the ambient temperature, very markedly influenced the behavioral threshold, the behavioral latency and the conduction velocity of the peripheral fibers but not the latency of the central decision-making.

**Conclusions/Significance:**

We have validated a psychophysical approach to nociceptive reactions for the mouse, which has already been described for rats and Humans. It enables the determination of four variables, which contribute to the overall latency of the response. The usefulness of such an approach was demonstrated by providing new fundamental findings regarding the influence of ambient temperature on nociceptive processes. We conclude by challenging the validity of using as “pain index" the reaction time of a behavioral response to an increasing heat stimulus and emphasize the need for a very careful control of the ambient temperature, as a prevailing environmental source of variation, during any behavioral testing of mice.

## Introduction

We recently developed in the rat, a psychophysical approach to nociceptive reactions which was based on the joint analysis of a thermal stimulus and the response [1]. The method was based mainly on random variations of the stimulus and the measurement of three observable variables, namely the initial temperature of the skin, the heating rate and the temperature reached at the actual moment of the reaction. This paradigm allows one to reach the two key descriptors of a behavioral response to noxious heat in psychophysical terms without using the reaction time (t_R_). These descriptors are the behavioral threshold (T_R_) and the behavioral latency (L_R_), both of which are latent variables which are inaccessible with conventional methods. Additional latent variables, e.g. the conduction velocity of the peripheral fibers that trigger the reaction and the latency of the central decision-making process, can be calculated using this approach.

We set aside the use of light bulbs because they give emissions in the visible and adjacent infrared parts of the electromagnetic spectrum, and since the skin is poorly absorptive and is reflective. In addition, by changing the voltage across the incandescent filament, one also changes the emission spectrum of an electric light bulb (see Figure 2C in [2]). Since the reflectance by, absorbance of and transmittance through, skin of incident radiation depends on its wavelength [3], a modification of the emission spectrum influences all the parameters of skin heating including the volume of tissue affected. This point is insurmountable when one wishes to vary the intensity of heat stimulation using light bulbs. To avoid such inconveniences of conventional methods of thermal stimulation, a CO_2_ laser stimulator was used and an infrared camera followed the resultant heating process.

The aim of the present study was to extend this approach to the mouse, a species that is more widely used for the development of pain tests or models (e.g. [4], [5]). The quantitative primary end-point for most behavioral tests of thermal nociception in the mouse, particularly in the so-called “tail-flick" test, is the reaction time (t_R_), i.e. the time lapse between the beginning of the application of heat and the evoked response. Since it is technically impossible to heat the skin instantaneously, the thermal stimulation is always progressive. The temporal background of such testing is therefore complex and it was the second aim of our study to clarify its construct validity in this species; in other words, to verify whether the “tail-flick" test and its variations effectively measure the targeted construct.

**Figure 1:**
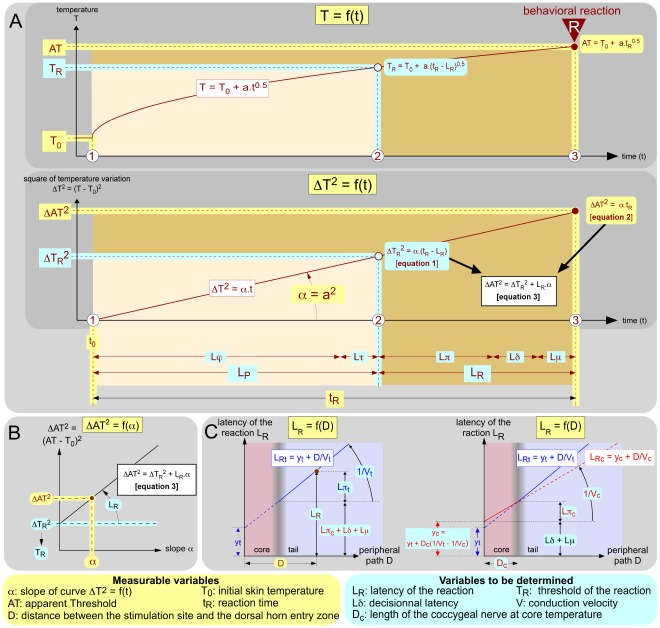
Measurable and calculated variables. The measurable variables are indicated with a yellow background and variables to be determined are indicated with a blue background. Abbreviations are available in Table 1. **A. When the skin is exposed to a constant source of radiant heat, the temperature T increases with the square root of time** from the initial temperature T_0_ according to the law of physics T = T_0_+a_*_T^0.5^ (upper graph) or, expressed in terms of squared temperature variations, ΔT^2^ = [T – T_0_)]^2^ = α_*_t (lower graph). When a behavioral reaction R occurs, the reaction time t_R_ is the sum of the time L_p_ it takes to warm up the skin up to the behavioral threshold T_R_ (i.e. the true nociceptive temperature threshold) plus the time L_R_ it takes for the reaction to occur once this threshold has been reached. The first period L_p_ belongs to the physical and biophysical domains, including heating (Lϕ) and transduction (Lτ) processes. During the second period L_R_, the temperature of the skin continues to increase up to the apparent temperature threshold AT reached at the time of the reaction. This second period is the latency of the reaction L_R_ that includes Lπ (the transit time for the spikes to reach the CNS), Lδ (the “decision" time required by the CNS for interpreting and processing this information for an order to be sent to the motor system) and Lμ (the time required for a motor response to be triggered). Overall, four variables are potentially accessible to experimental measurement: T_0_, AT, t_R_ and α (yellow background). The heating process can be described by three key moments: 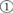 the beginning of stimulation, t = t_0_ and T = T_0_; 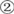 the moment of the triggering of the reaction defined by ΔT_R_^2^ = (T_R_ – T_0_)^2^ = α_*_(t_R_ – L_R_) [**equation 1**]; 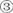 the moment of the reaction defined by ΔAT^2^ = (AT – T_0_)^2^ = α_*_t_R_ [**equation 2**]. B. The linear relationship ΔAT^2^ = ΔT_R_^2^+L_R*_α [equation 3] is obtained by substituting α_*_t_R_ of equation 2 into equation 1 and is used for extraction of the two variables to be determined (blue background): the intercept and the slope of this linear function represent ΔT_R_^2^ and L_R_, respectively (see Fig. 4B). **C.** **Theoretical relationship between the distance D, separating the site of stimulation on the tail from the dorsal root entry zone, and the latency of the behavioral reaction L_R_**. The available experimental data from the tail at T_0_ are shown as a blue line (left graph). The reciprocal of the slope of this line corresponds to the conduction velocity V_t_ of the fibers that triggered the reaction. However, the conduction velocity of these fibers increases when the coccygeal nerves travel through the core of the animal, which is set at T_c_ by thermoregulatory processes. This second component of the peripheral process is shown in red (right graph). The latency of these two components is Lπ = Lπ_t_+Lπ_c_ = D_t_/V_t_+D_c_/V_c_. The intercept y_c_ = y_t_+D_c*_(1/V_t_ – 1/V_c_) of the red straight line with the ordinate represents the part of L_R_ that does not deal with the peripheral processes, i.e. Lδ+Lμ.

As with any psychophysical process [6], [7], a nociceptive response produced by a radiant source of heat results from a series of events, each with its own duration. The reaction time (t_R_) is thus the sum of sequential physical/biophysical (L_P_) and behavioral/psychophysical (L_R_) serial latencies (Figure 1A). Starting from an initial temperature (T_0_), the stimulus increases during L_P_ to reach the threshold of the reaction (T_R_); the reaction is triggered following the response latency (L_R_). L_P_ is the sum of: (1) the time (Lϕ) required to reach the threshold of nociceptors and (2) the time (Lτ) required for transduction. L_R_ is the sum of: (1) the peripheral latency (Lπ) required for the nociceptive information to reach the CNS; (2) the ‘decisional’ latency (Lδ) required for the central decision-making process, initiated by the arrival (and/or the accumulation) in the CNS, of a sufficient amount of nociceptive information to trigger the behavioral reaction; and (3) the motor latency (Lμ) time from motoneuron activation up to the shortening of the muscles. The central decision-making process must be understood in its most general sense, i.e. as a distributed neural network that receives and handles afferent sensory information and issues an order to the relevant muscle groups that effects a response [7].

**Table 1:** Symbols, abbreviations and units.

In Figures	
Yellow background	related to variable measured experimentally
Blue background	related to latent variable to be determined
Brown	related to individual curves of interest
**In Text and Figures**	
a	Slope of the heating ramp = α^0.5^ (°C/s)
α	Slope of the squared temperature variation (°C^2^/s) = a^2^
AT	Apparent threshold (°C)
CNS	Central nervous system
D	Distance between the stimulation site and the dorsal horn entry zone (mm) = D_t_+D_c_
D_c_	Distance between the tail-trunk interface and the dorsal horn entry zone (mm)
D_t_	Distance between the stimulation site and the tail-trunk interface (mm)
ΔAT	Temperature variation between the initial temperature and the apparent threshold (°C) = AT – T_0_
ΔT	Temperature variation with reference to the initial temperature (°C) = T – T_0_
K	Composite constant grouping together the biophysical properties of skin
k	Thermal conductivity = 0.696 (W_*_m^-1^_*_°C^-1^)
L	Latency (ms) = unobserved hypothetical time
Lδ	Decisional latency (ms) = time required by the CNS for interpreting and processing the nociceptive information
Lμ	Motor latency (ms) = time from motoneuron activation up to the shortening of the muscle.
Lπ	Peripheral latency (ms) = transit time for spikes in primary afferents to reach the entry zone to the spinal cord = Lπ_t_+Lπ_c_
Lπ_c_	Peripheral latency within the core (ms) = transit time for spikes in primary afferents between the tail-trunk interface and the dorsal horn entry zone
Lπ_t_	Peripheral latency within the tail (ms) = transit time for spikes in primary afferents between the stimulation site and the tail-trunk interface
L_R_	Latency of the reaction (ms) = time period which separates the moment at which T_R_ is reached from the actual moment of the behavioral response R
Lφ	Physical latency (ms) = duration of the skin heating process from the initial skin temperature T_0_ to trigger transduction in nociceptors
Lτ	Transduction latency (ms) = time required for heat to be transduced by nociceptors into neuronal spikes
L2	Second lumbar vertebra
L4	Fourth lumbar vertebra
MPE	Maximum possible effect = 100_*_(reaction time – control reaction time)/(cut-off time – control reaction time)
q	Laser power (mW)
Q	Density of laser power (mW/mm^2^)
Q_10_	Ratio of the conduction velocity at one temperature to the conduction velocity at a temperature 10°C colder.
R	Behavioral response
S	Stimulation surface area (mm^2^)
t	Time (ms)
t_0_	Beginning of the stimulation
t_R_	Moment of the behavioral response = reaction time (ms)
T	Skin temperature (°C)
T_a_	Ambient temperature (°C)
T_c_	Core temperature (°C)
T_max_	Maximal skin temperature, achieved following a laser stimulus (°C)
T_0_	Initial skin temperature (°C)
T_R_	Threshold of the reaction (°C)
TFL	Tail-flick “latency" = t_R_/1000 (s)
V	Conduction velocity (m/s)
V_c_	Conduction velocity of fibers traveling in the core that trigger the behavioral response (m/s)
V_t_	Conduction velocity of fibers traveling in the tail that trigger the behavioral response (m/s)
y_c_	Intercept of the straight line L_R_ = f(D) with the ordinate, calculated for the central temperature T_c_ (ms) = y_t_+D_c*_(1/V_t_ – 1/V_c_) = Lδ+Lμ
y_t_	Intercept of the straight line L_R_ = f(D) with the ordinate, calculated for the basal temperature of the tail T_0_

We will use the term “apparent threshold" (AT) for the skin temperature reached at the end of the sequence of latencies summing up to t_R_. At the time when the reaction threshold (T_R_) level of stimulation is achieved, i.e. time L_P_ = t_R_ – L_R_, the flow of information transmitted by the nociceptors is sufficient to trigger the response at time t_R_.

Following an evaluation of the potential variations of the temperature of the tail in absence of any other manipulations, we used such analyses to determine the latent behavioral variables T_R_ and L_R_ in the mouse (Figure 1B). The investigation of the influence of both the stimulation site and the ambient temperature on these dependent variables led to an estimation of the conduction velocity of the fibers and the time required for the central decisional processes that triggered the reaction (Figure 1C). Finally, we propose, and have verified experimentally, a simple model for computing the variations of t_R_, the so-called ‘tail-flick latency’ (TFL), elicited by changes in either the power of a radiant heat source, the initial temperature of the skin or the site of stimulation on the tail.

## Results

First, we will present a preliminary set of experiments designed to evaluate the potential variations of the temperature of the tail in the absence of any other manipulations. Then we will detail an individual example of the determination of behavioral thresholds and latencies. The following sections will provide general data regarding the role of the stimulation site and the ambient temperature on these dependent variables, leading to considerations on the conduction velocity of the fibers and the central processes (decisional latency) that trigger the reaction. Finally, we will propose, and verify experimentally, a simple model for computing the variations of t_R_, the so-called ‘tail-flick latency’ (TFL), elicited by changes in either the power of the radiant heat source, the initial temperature of the skin or the site of stimulation on the tail.

### Spontaneous tail temperature variations

**Figure 2:**
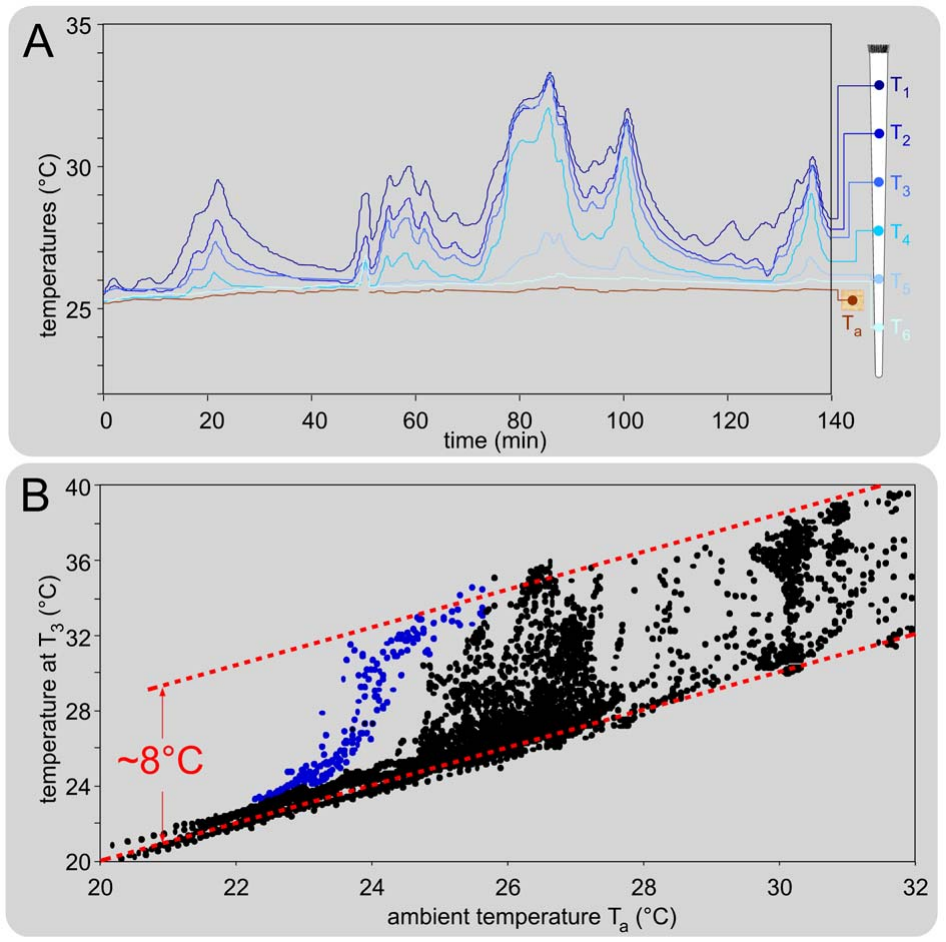
Spontaneous variations of the temperature of the tail. **A. Individual example** of the concomitant recordings at 1 Hz of the ambient temperature T_a_ and 6 points spread out along the rostro-caudal extent of the tail (T_1_-T_6_, shown on the drawing on the right). Note the spontaneous fluctuations of the temperature (maximum 8°C) of the proximal two-third of the tail. **B. Relationship between the ambient temperature T_a_ and the temperature of the tail at point T_3_.** Overall effects obtained in 15 mice placed in various ambient temperatures in the 20-32°C range. The blue points were obtained from sequences during which the ambient temperature decreased and included active thermoregulation processes (see an example in Figure 9).

The temporal evolution of the ambient and tail temperatures was recorded using a thermometric camera, at a rate of 1/s in a preliminary series of experiments in conditions similar to those used for behavioral testing. An example is shown in Figure 2A where the room temperature increased slowly: below ∼25°C (not shown), the temperature of the tail was essentially similar to the ambient temperature but above 25°C, there were spontaneous fluctuations of the temperature of the proximal two-thirds of the tail, at a rate of ∼2-3 cycles per hour. Such recordings were replicated in 15 mice with ambient temperature in the 20-32°C range. Figure 2B shows the overall relationship between the ambient temperature T_a_ and the temperature of the tail at point T_3_. Below 24°C T_a_, the skin temperatures were close to T_a_, revealing a complete vasoconstriction state in all the mice tested. Above 25°C T_a_, the temperature fluctuated in most animals. Above 30°C T_a_, the skin temperature was generally several degrees higher than T_a_. The envelop of the points in the ambient-tail temperatures plane, reveals spontaneous variations of up to 8°C of the tail when ambient temperature was above 25°C. Dynamic 8°C-oscillations, as exemplified in Figure 2A, indicated the thermo-neutral state of the mouse.

### Behavioral threshold and behavioral latency: an individual example

**Figure 3:**
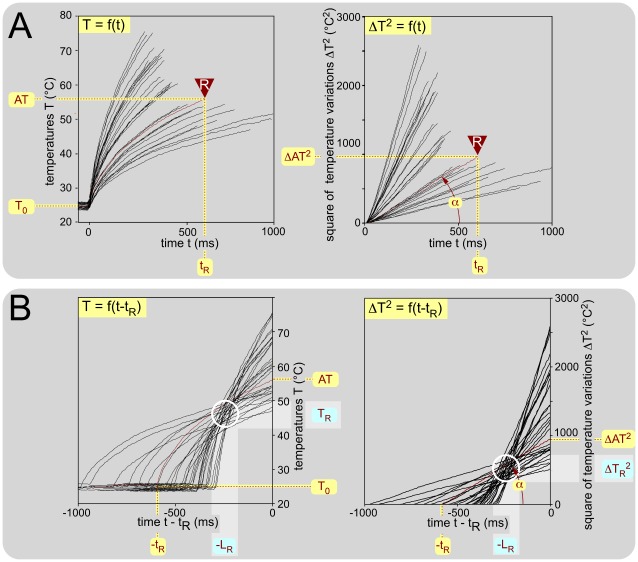
Example from an individual animal, of behavioral responses elicited by stimulation of the middle of the tail. The distance D from this site to the entry zone in the cord was 115 mm and the stimulus (70-320 mJ) was applied from time 0 until the movement of the tail. The mean basal temperature T_0_ was 24.7 (24.5-25.0)°C. **A. Temporal evolution of the temperature of the skin** (31 trials). Left graph: temporal evolution of the temperature of the skin T = f(t) recorded in the center of the heating spot. Right graph: Identical data expressed in terms of squared temperature variations. All these linear relationships were highly significant and their slopes α could therefore be computed confidently. Each individual trial was fully summarized by four measures, namely T_0_, AT, t_R_ and α. These measures were used for building the black graphs in Figure 4B-D. **B. Changing the origin of time.** When one changes the origin to center the heating curves on the actual moment of the reaction, one can visualize the temporal evolution of the sequence of the preceding events either in terms of temperature (left graph) or square of temperature variation (right graph). Note the clear tendency of these curves to cross each other in a privileged zone (open white circle; see text).

The left part of Figure 3A shows a series of heating curves for the tail obtained from recordings through the thermometric camera at 100 Hz. The recordings were triggered 350 ms before the application of a constant power laser infrared radiation to the middle part of the tail. The recordings were stopped by the tail withdrawal. As expected, the temperature increased with the square root of time, according to the law of radiant heat transfer T = T_0_+a_*_t^0.5^ [8]. Expressed in terms of squared temperature variations, these relationships became linear in t: ΔT^2^ = a^2^_*_t = α_*_t (Figure 3A right). Since T_0_ remained stable during the experimental procedure, we could infer T_R_ and L_R_, the threshold temperature and latency of the response - which are presumably constant - from a series of trials where the power of the radiant heat source varied to produce an appropriate range of α (here 0.6-10°C^2^/ms). By adjusting the origin of the time scale of each individual heating curve to the actual time of the reaction, one can visualize the back-timing of events. In this temperature-time plane, each trajectory crosses every other (at a communal point) within a bounded region that allows the determination of T_R_ and L_R_ (Figure 3B).

From an experimental standpoint, each behavioral trial can be summarized by four accessible quantities measured independently, namely: the initial skin temperature (T_0_), the apparent threshold (AT), the reaction time (t_R_) and the slope (α). From the theoretical standpoint, the behavioral process can be described by three key moments: (1) the beginning of stimulation, t = t_0_; the moment of the triggering of the reaction defined by t = t_R_ – L_R_ and T_R_ = T_0_+a_*_(t_R_ – L_R_)^0.5^; (3) the moment of the reaction defined by t = t_R_ and AT = T_0_+a_*_t_R_^0.5^. For a given initial skin temperature T_0_, one can consider the squared temperature variations (ΔT^2^), yielding the following expressions: ΔT_R_^2^ = α_*_(t_R_ - L_R_) [**equation 1**] and ΔAT^2^ = α_*_t_R_ [**equation 2**]. One can then substitute the α_*_t_R_ of equation 2 in equation 1 to obtain a linear relationship: ΔAT^2^ = f(α): ΔAT^2^ = ΔT_R_^2^+L_R*_α [**equation 3**].

**Figure 4:**
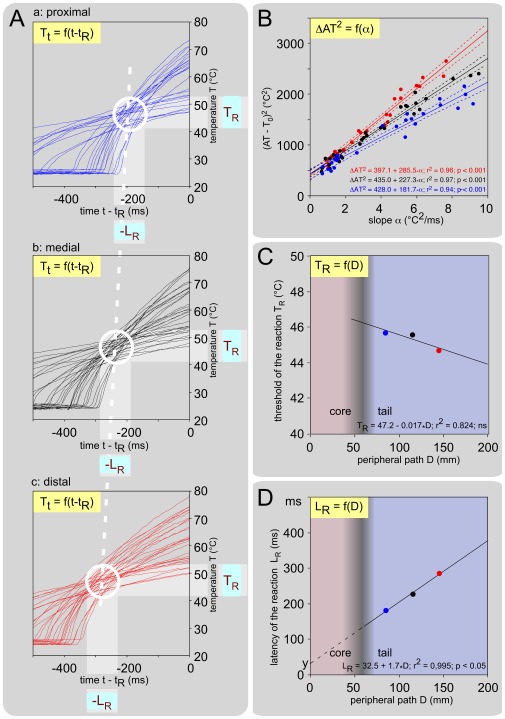
Individual example of the behavioral responses elicited by stimulation at three rostro-caudal levels on the tail, 25 mm apart. The mean temperature of the tail was 24.8 (24.6-25.0)°C. **A. Temporal evolution of the temperature of the skin during the application of various powers of stimulation** (70-320 mJ) with the time origin being adjusted to the actual moment of triggering of the reaction, T = f(t – t_R_), recorded in the center of the heating spot applied on the proximal (a: 25 trials), medial (b: 31 trials) and distal (c: 28 trials) parts of the tail. Note the clear tendency of these curves to cross each other in a privileged zone (open circles) and the progressive shift of this zone backward in time when the stimulation site moved from proximal to distal parts of the tail (white dashed line). **B. Relationships between the apparent threshold AT and the slope α**, ΔAT^2^ = f(α), calculated for the 3 sites of stimulation. The strong linear relationships provided accurate calculations of T_R_ and L_R_ for each level of stimulation (dotted lines: ±95% CI). **C. Relationship between the calculated behavioral threshold T_R_ and the distance D** that separated the site of stimulation on the tail from the entry zone in the cord, as obtained from data shown in B. **D. Relationship between the calculated behavioral latencies L_R_ and the distance D** that separated the site of stimulation on the tail from the entry zone in the cord, as obtained from data shown in B. L_R_ was directly proportional to D. The reciprocal of the slope represents the conduction velocity (0.59 m/s) of the fibers that triggered the reaction, in that part of their course which is within the tail.

The experimental data fully verified this theoretical statement. Figure 4 extends the individual example shown in Figure 3 by stimulating two additional parts of the tail, 25 mm proximal and distal from the stimulation site already shown. The mean basal temperature of the skin (T_0_) was 24.8 (24.6-25.0)°C. In Figure 4A the origin of the time scale of each individual heating curve is adjusted to the actual time of the reaction. In such a back-timing analysis, the bounded region where each trajectory crosses the others, moved to the left when the stimulation site was displaced backward on the tail (Figure 4A, top to bottom). The three corresponding linear relationships ΔAT^2^ = f(α) were highly significant (Figure 4B), providing the numerical values of ΔT_R_^2^ and L_R_ for each point of stimulation. Since T_0_ was stable, T_R_ = (ΔT_R_^2^)^ 0.5^ – T_0_. Note that the two key descriptors of the behavioral response to noxious heat, namely T_R_ and L_R_, were determined without any use of t_R_, as each individual response was fully described by α, T_0_ and AT. The distance D between the stimulation site on the tail and the dorsal horn entry zone in the spinal cord, appeared to be a major source of variation. Indeed, the two descriptors changed when the stimulation site along the tail was moved backwards: T_R_ decreased (Figure 4C) and L_R_ increased (Figure 4D). The inverse of the slope of the L_R_ = f(D) relationship allowed the calculation of the conduction velocity of the fibers that triggered the reaction, at least in the part of their course within the tail: V_t_ = 1/1.7 = 0.59 m/s.

### Role of the stimulation site and conduction velocity of the fibers that triggered the reaction

**Figure 5:**
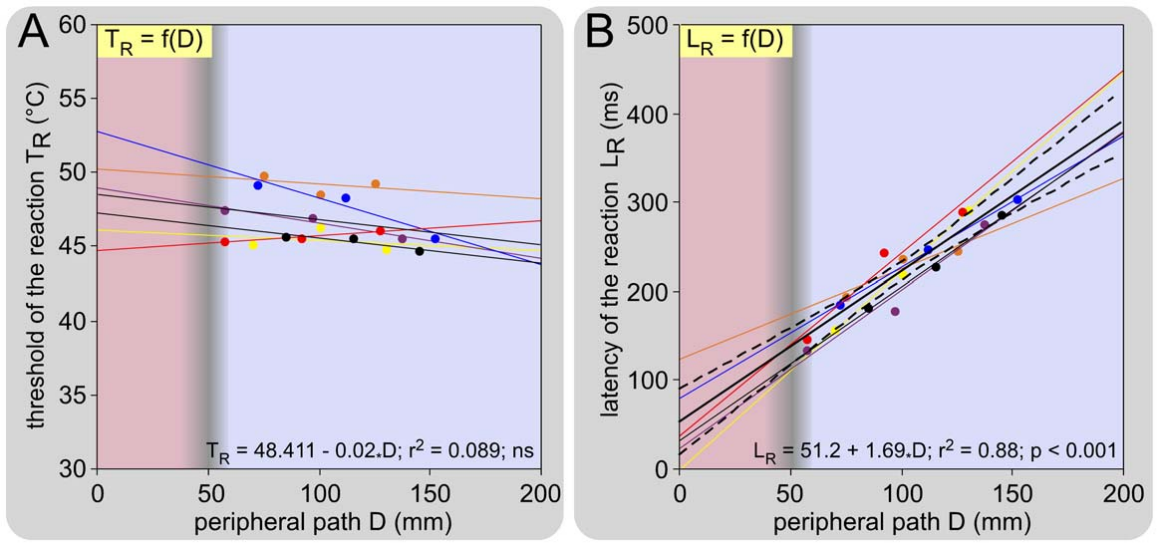
Overall influence of the levels of stimulation on behavioral thresholds and latencies. Observations were made on 6 mice with 3 stimulation sites. The mean basal temperature of the skin T_0_ was 25.6 (25.0-26.1)°C. **A. Relationships between the calculated behavioral threshold T_R_ and the distance D** that separated the site of stimulation on the tail from the entry zone to the cord. On average, the behavioral threshold T_R_ was 46.7 (45.9-47.5)°C with a non-significant tendency to decrease from the proximal to the distal parts of the tail. **B. Corresponding relationships between the calculated behavioral latencies L_R_ and the distance D** that separated the site of stimulation on the tail from the entry zone in the cord (individual and overall regression lines are shown as fine and large lines, respectively). Overall, there was a very significant linear relationship between D and L_R_ (L_R_ = 51.2+1.69_*_D; F_1-16_ = 117.6; p<0.001; dotted lines: ±95% CI). The inverse of the slope of these relationships allowed the calculation of the conduction velocities of the fibers that triggered the reaction: 0.62 (0.47-0,78) m/s.

The experiments detailed above were reiterated on 8 mice in a ∼25°C room and the mean basal temperature of the skin T_0_ was 25.6 (25.0-26.1)°C. This temperature was stable during a session. In every case, the classical method for calculating nerve conduction velocities was used by changing the distance D between the stimulation site on the tail and the dorsal horn entry zone in the spinal cord. On average, the threshold T_R_ was 46.7 (45.9-47.5)°C, but displayed a tendency to get lower for distal sites of stimulation in the 44.7-49.8°C range (Figure 5A). L_R_ was directly proportional to D (Figure 5B). The reciprocal of the slope represents the conduction velocity of the fibers that triggered the reaction, in the part of their course that is within the tail. The parallelism of the relationships L_R_ = f(D) suggests homogeneity across animals in respect of the nerve fibers implicated in the reaction. The mean conduction velocity of these fibers in the section of their course traveling in the tail was 0.62 (0.47-0.78) m/s, thus categorizing them as unmyelinated C-fibers. We did not see any evidence that Aδ-fibers participate to the triggering of the tail withdrawal.

### Ambient temperature as a key factor of variations

**Figure 6:**
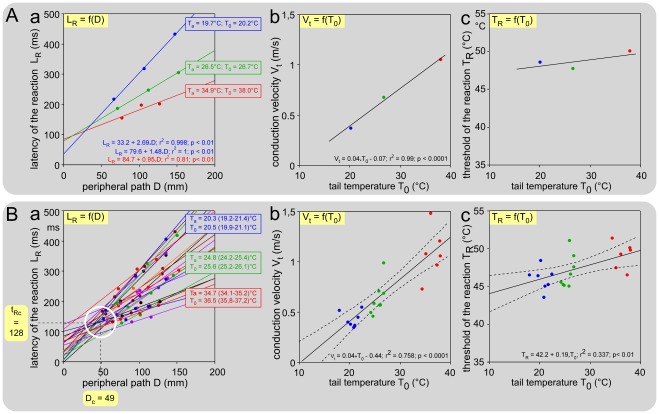
Effects of the ambient temperature on the behavioral responses. Ambient temperatures were ∼20°C (blue), ∼25°C (green), ∼35°C (red). **A. An individual example** from a mouse tested three days apart. The L_R_ = f(D) plots shown in (a) reveal a decrease of the slope when the temperature increased, witness of the increased conduction velocity, itself shown in (b) by the V_t_ = f(T_0_) plot. The effects of the ambient temperature on the behavioral threshold are shown in (c) in terms of T_R_ = f(T_0_) plot. **B. Corresponding overall effects** from 8 mice. (a) Bundle of overall L_R_ = f(D) straight lines obtained with various basal temperatures T_0_. Note that these lines tend to cross each other in a privileged zone (white open circle) corresponding to the tail-trunk interface where the temperature of the nerves increases from T_0_ to the core temperature T_c_. The highest density of intersections was located at coordinates D_c_ = 49 mm and t_Rc_ = 128 ms. (b) V_t_ = f(T_0_) plot. A highly significant linear relationship was seen: V_t_ = 0.04_*_T_0_ – 0.44 (F_19,1_ = 59.5; p<0.01; dotted lines: ±95% CI). A mean Q_10_ = 2.1 (1.9-2.4) between 20 and 30°C was calculated. (c) T_R_ = f(T_0_) plots. A significant linear relationship was seen: T_R_ = 42.2+0.19_*_T_0_ (F_19,1_ = 9.7; p<0.01; dotted lines: ±95% CI). This indicates that overall the threshold T_R_ increased as the temperature of the skin increased.

We replicated the experiments with mice being introduced in a chamber where the ambient temperature was maintained stable during a given session, but changed over sessions in the 18-35°C range. For each basal skin temperature, the corresponding ΔAT^2^ = f(α) plots allowed calculations of L_R_ and T_R_ for each level of stimulation on the tail. Mice were submitted to three ambient temperatures, as shown with individual example in Figure 6Aa (mean T_a_ = 19.7, 26.5 and 34.9). Several points should be pointed out: (1) The basal skin temperature (T_0_) was close to the ambient temperature (T_a_) for the two colder environments but 3°C above for the warmer. (2) The slope of the L_R_ = f(D) functions fell as the tail temperature increased, which was testimony to the increased conduction velocities of the fibers that triggered the behavioral reaction (Figure 6Ab). (3) The mean thresholds were roughly similar (Figure 6Ac).

The overall results are summarized in Figure 6B where the experiments are categorized and colored in three groups according to the ambient temperature T_a_ (blue ∼20°C, green ∼25°C, red ∼35°C). In all these cases, the temperature was homogenous along the length of the tail. In the 27-34°C range, it was not possible to calculate any conduction velocity because of gradients in temperature along the tail. The L_R_ = f(D) lines tend to cross each other in a privileged zone (white open circle) corresponding to the tail-trunk interface where the temperature of the nerves increases from T_0_ to the core temperature T_c_ (Figure 6Ba). Analyzing in terms of density, the cluster of the intersection points of each straight line with the others in the D-L_R_ plot, revealed that the highest density of intersections was located at coordinates D_c_ = 49 mm and t_Rc_ = 128 ms. The overall relationships of V_t_ = f(T_0_) revealed a linear function best described by the equation V_t_ = 0.04_*_T_0_ – 0.44, with Q_10_ between 20 and 30°C = 2.0 (1.9-2.4) (Figure 6Bb). Finally, the calculations revealed that the behavioral threshold T_R_ increased slightly but significantly as the skin temperature T_0_ increased - this was best described by the equation T_R_ = 0.19_*_T_0_+42.2 (Figure 6Bc). As grand-mean, the behavioral threshold was T_R_ = 47.3 (46.4-48.3)°C.

We wish emphasize here that L_R_ was always less than 500 ms, even, remarkably, in the cases where the extremity of the tail was stimulated in a cold environment a situation that elicited the tardiest responses (see the right hand higher corner of the graph shown in Figure 6Ba). In those cases, T_c_ declined [35.5 (36.5-34.5)°C at 90 min].

### Decisional latency

The distance D_c_ corresponds to an approximation of the interval between the tail-trunk interface, where the conduction velocity increases, and the dorsal horn entry zone. At this point, the temperature of the nerve increased from the temperature of the tail T_t_ to the core temperature T_c_. The reasonable assumption that the effect of temperature remains constant along the length of the fibers, allows the calculation of the conduction velocity of fibers at core temperature in the trunk: V_c_ = V_t_+0.04_*_(T_c_ – T_0_). Note incidentally that t_Rc_ provides an estimation of the sum of several latencies: Lπ_c_ (peripheral process within the core), Lδ (decisional) and Lμ (motor).

The availability of both V_c_ and D_c_ provides keys for an estimation of the decisional latency Lδ to be inferred from the intercept y_c_ of the regression line L_R_ = f(D) with the ordinate. Because of the increased conduction velocity within the core, the intercept y_t_ should be corrected as follow: y_c_ = y_t_+D_c*_(1/V_t_ – 1/V_c_). The corrected intercept y_c_ represents the latencies not related to peripheral events, namely the decisional and motor latencies (Lδ+Lμ). Here we calculated the mean y_c_ = 91 (78-103) ms. Lμ is unknown in mice but was calculated for the tail-flick response in rats by Danneman et al. [9]: Lμ = 4 ms. Knowing the quickness of motor efferent fibers and the sizes of the animals, we considered that Lμ could not be longer in mice. In summary, the decisional latency was on average 87 (74-99) ms. There was no significant correlation between the individual central decision-making latencies and the ambient or skin temperature.

### Further determination of the type of fiber that trigger the reaction

The conduction velocity of the peripheral fibers responsible for the withdrawal corresponds to C-fibers. Since in several species, the threshold of activation of individual nociceptors is higher for Aδ- than for C-fibers [10]-[12], we could not exclude the possibility that higher stimuli would have been able to activate Aδ-fiber nociceptors in mice. High power (α = 80°C^2^/ms; T_max_ ∼70°C) but very short laser pulses (30 ms) of heat were therefore applied to various sites along the tail and the corresponding reaction times measured. If the two types of fibers were involved in triggering such reactions, it was assumed that the reactions recorded would be elicited by the faster fibers, namely the Aδ-fibers.

**Figure 7:**
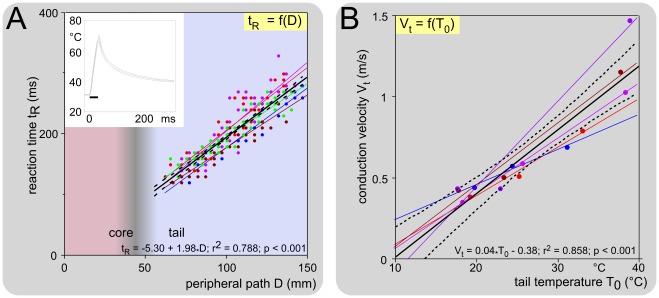
Reaction time of response elicited by high-power, short-duration, laser pulses. An example of recording of the mean temperature (±95% CI) of 10 pulses is shown in the insert (α = 80°C^2^/s; T**_max_** ∼70°C; duration 30 ms). **A. Example of the linear relationship between the distance D, separating the site of stimulation on the tail from the dorsal root entry zone, and the reaction time** in cases of similar temperature of the skin: T_0_ = 24.5 (23.4-25.5)°C. The relationships t_R_ = f(D) were very significantly linear and parallel, suggesting homogeneity along the tail and across animals of the conduction velocities of the peripheral fibers that triggered the reaction. The inverse of the slope of these straight lines represents the conduction velocity of these fibers, in that part of their course which is within the tail: 0.52 (0.47-0.57) m/s. **B. Corresponding V_t_ = f(T_0_) plot.** Note the highly significant linear relationship, best described by the equation V_t_ = 0.04_*_T_0_ – 0.38.

Figure 7A shows a series of such experiments made at an ambient temperature of ∼24°C. The linear relationships t_R_ = f(D) were very significant and parallel, suggesting homogeneity along the tail and across animals in respect of the conduction velocities of the peripheral fibers that triggered the reaction. The mean conduction velocity of these fibers in the part of their course within the tail, calculated by this approach, was 0.52 (0.47-0.57) m/s. The unmyelinated nature of the fibers that trigger the behavioral reaction was therefore confirmed in the experimental conditions with short-duration, high-intensity stimuli.

We also replicated the preceding experiments with mice being introduced to a chamber in which the ambient temperature changed over sessions in the 18-34°C range (Figure 7B). As expected, the slopes of the regression lines of the relationships t_R_ = f(D) increased when temperature of the tail decreased. In other words, the warmer the tail, the faster the conduction velocity. This resulted in a highly significant linear relationship between the temperature of the tail and the conduction velocity, best described by the equation V_t_ = 0.04_*_T_0_ – 0.38, with Q_10_ = 2.0 (1.6-2.5) between 20 and 30°C. Note the striking similarity of the graphs shown in figures 7B and 6Bb.

### Modeling and simulation of the “tail-flick latency"

The possibility exists to compute the variations of the reaction time t_R_ (e.g. the so-called ‘tail-flick latency’, TFL), elicited by changing any of the parameters. One can summarize the data described above as follows: t_R_ = L_P_+L_R_ = (T_R_ – T_0_)^2^/α+L_R_ = (T_R_ – T_0_)^2^/α+Lπ+Lδ+Lμ = (T_R_ – T_0_)^2^/α+D_t_/V_t_+D_c_/V_c_+Lδ+Lμ = (T_R_ – T_0_)^2^/α+(D – D_c_)/V_t_+D_c_/V_c_+Lδ+Lμ. Taking into account this relation and (1) the threshold of the reaction, T_R_ = 42.2+0.19_*_T_0_, (2) the conduction velocity of the fibers within the tail of the animal, V_t_ = 0.04_*_T_0_ – 0.44, (3) the length, D_c_ = 49 mm of the coccygeal nerve traveling within the core, (4) the corresponding conduction velocity of the fibers, V_c_ = 0.04_*_T_c_ – 0.44, (5) Lδ+Lμ = 91 ms, one can obtain after rearrangement: t_R_ = f(α, T_0_, T_c_, D) = (42.2 – 0.81_*_T_0_)^2^/α+(D – 49)/(0.04_*_T_0_ – 0.44)+49/(0.04_*_T_c_ – 0.44)+91.

We also computed the ambient and core temperatures and found a very significant polynomial regression: T_c_ = 20.5+1.017_*_T_a_ – 0,0139_*_T_a_^2^ (F_2,33_ = 174,4; p<0.001), which was witness to a torpor state of the animal confronted by a cold environment. This expression was introduced into the preceding equation for the calculation of t_R_ = f(α, T_0_, T_a_, D). However, because of the very low impact of this factor of variation on t_R_ (<2% at the most), all calculations were simplified with T_c_ = 38°C. Computation of the reaction time is summarized as t_R_ = f(α, T_0_, D) = (42.2 – 0.81_*_T_0_)^2^/α+(D – 49)/(0.04_*_T_0_ – 0.44)+137.

**Figure 8:**
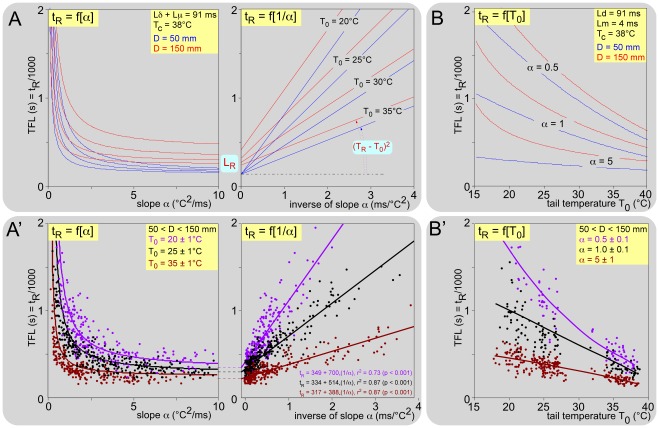
Modeling and simulation of the tail-flick “latency". t_R_ = (T_R_ – T_0_)^2^/α+(D – D_c_)/V_t_+D_c_/V_c_+Lδ+Lμ. Taking into account the following relationships T_R_ = f(T_0_), V_t_ = f(T_0_), V_c_ = f(T_c_) and the length D_c_ = 49 mm, one obtain: t_R_ = f(α, D, T_c_, T_0_) = (42,17 – 0.81_*_T_0_)^2^/α+(D – 49)/(0.04_*_T_0_ – 0.44)+49/(0.04_*_T_c_ – 0.44)+Lδ+Lμ (see text). **Upper graphs (A, B)**: theoretical curves; the blue and red lines are computations for stimulation of two sites on the tail, proximal and distal, 50 and 150 mm from the dorsal root entry zone, respectively. **Lower graphs (A’, B’)**: corresponding experimental data obtained from eight mice. The numerical values of the parameters used in the equation are shown in the inserts. **A. Results of simulations of the variations in the reaction time (t_R_ or TFL) introduced by varying the power of a radiant heat source (α varying).** As expected from the form of the equation, the t_R_ = f(α) computation produced hyperbolas, with the horizontal asymptote representing L_R_ (left graph). The right graph shows the relation t_R_ = f(1/α), which transforms this curve into a linear relationship. The slope and the intercept with the ordinate of the straight line represent (T_R_ – T_0_)^2^ and L_R_, respectively. These computations were made for four temperatures of the tail: 20, 25, 30 and 35°C, the last being achieved when the animal dissipates heat by vasodilatation of the tail for any reason. **B. Role of basal skin temperature (T_0_ varying):** theoretical curves made for three α values: 0.5, 1 and 5. **A’. Role of heating power (α varying): experimental data** and corresponding regression lines obtained from 8 animals are presented for three ranges of skin temperatures T_0_, namely 20 (violet), 25 (black) and 35°C (brown). The respective regression equations are shown as an insert. **B’.** **Role of basal skin temperature (T_0_ varying): experimental data** and corresponding regression lines from the same animals are presented for three ranges of heating rate expressed as α, namely 0.5 (violet), 1 (black) and 5 (brown). For an averaged distance D = 100 mm, the reaction time was computed as t_R_ = f(α, T_0_) = 0.656/α_*_T_0_^2^ – 34.2/α_*_T_0_+(1781+136.37α)/α+1275/(T_0_ – 1.1). The parameters and coefficients of regression were then estimated by a nonlinear least squares fit to the data in the form t_R_ = a_*_T_0_^2^ – b_*_T_0_+c+d/(T_0_+e): t_R_ = 2.37_*_T_0_^2^ – 209.64_*_T_0_+4943+208.7/(T_0_ – 27.9); t_R_ = 0.037_*_T_0_^2^ – 42.03_*_T_0_+1844 – 151.3/(T_0_ – 22.5); t_R_ = – 0.223_*_T_0_^2^ – 3.02_*_T_0_+613 – 35.7/(T_0_ – 23.9); for α = 0.5, 1 and 5, respectively (r^2^ = 0.878, 0.686 and 0.661 respectively; p<0.001 for all).

In the classical tail-flick test, the principal source of variation introduced by experimenters is the thermal radiation emitted by the electrical bulb used for achieving a predetermined range of TFL values. This corresponds here to variations in the parameter α. As expected from the relation t_R_ = ΔT_R_^2^/α+L_R_, such computation produced hyperbolae when the basal temperature was stable (Figure 8A, left graph), with the horizontal asymptote representing L_R_. A simple anamorphous transformation [t_R_ = f(α^-1^)] linearized this relationship (Figure 8A, right graph). The slope and the intercept with the ordinate of the straight line represent (T_R_ – T_0_)^2^ and L_R_, respectively. The latter is the reaction time that one would expect following instantaneous heating (α → ∞). Note the low impact of the stimulation site (or peripheral conduction distance) and the very high impact of the basal skin temperature. This can be also seen with the t_R_ = f(T_0_) relationships, computed for several powers of heating (Figure 8B).

These theoretical relationships were verified by using the 1650 recordings of individual reactions of the mice, in terms of t_R_ = f(α) and t_R_ = f(1/α) for various basal temperatures and in terms of t_R_ = f(T_0_) for various heating ramps. The linearity of the relations t_R_ = f(1/α) is illustrated for three basal temperatures, namely 20, 25 and 35°C (Figure 8A’). The relations t_R_ = f(1/T_0_) is illustrated for three ranges of heating rate expressed as α, namely 0.5, 1 and 5 in Figure 8B’. For an averaged distance D = 100 mm, computation of the reaction time can be written t_R_ = f(α, T_0_) = 0.656/α_*_T_0_^2^ – 34.2/α_*_T_0_+(1781+136.37α)/α+1275/(T_0_ – 1.1). The parameters of this equation were estimated by the method of least squares.

**Figure 9:**
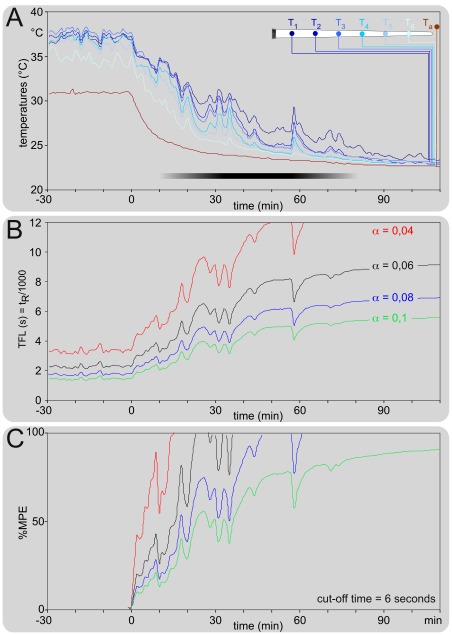
Modeling and simulation of the tail-flick “latency" in a concrete situation of ambient temperature variation. **A. Individual example of the concomitant recordings at 1 Hz of the ambient temperature T_a_ and 6 points spaced out along the rostro-caudal extent of the tail of a mouse** (T_1_-T_6_, shown on the drawing on the right hand upper corner). The ambient temperature T_a_ was stable during a 30-minutes controlled warm period and then fell. The tail was vasodilated during the control period and then declined in three successive phases: passive diminution, active regulation (black horizontal bar) and vasoconstriction, with all temperatures finally converging toward T_a_. **B. Theoretical computed calculation of the TFL** (t_R_), elicited from point T_3_ for several powers of heating (indicated on the right). **C. Identical calculation but expressed if terms of percentage of “maximum possible effect"** [13]: %MPE = 100_*_(reaction time – control reaction time)/(cut-off time – control reaction time). Here, the control reaction time was the mean TFL calculated during the 30-minutes warm period and the cut-off time was 6 seconds. Note the dramatic consequences of any T_a_ changes.

In order to simulate these relationships in a more palpable or tangible fashion, we used one of the experiments used for building Figure 2B, where the ambient temperature T_a_ was warm during a 30-minute stable control period, and then declined. The evolutions of the tail and ambient temperatures are shown in Figure 9A. The control period is characterized by a strong vasodilatation with the temperature of the tail being 7-8°C above T_a_ except for the distal part which was slightly colder. Then, one sees three successive phases: passive decline, active regulation and vasoconstriction. The computed calculation of t_R_, or tail-flick “latency" (TFL) elicited from the middle of the tail (point T_3_) is shown in Figure 9B for several powers of heating (0.04<α<0.1) chosen because they led to a 2-4 seconds TFL in the control period. Note the large, power-dependent, increases of TFL. Such variations can be amplified by the so-called maximal possible effect (MPE, Figure 9C), often used in pharmacological experiments, as initially proposed by Harris and Pierson [13]. In summary, a huge impact of the temperature of the skin was seen on these variables.

## Discussion

We have validated in the mouse, a psychophysical approach to nociceptive reactions which had been described already in both rats and human beings [1], [14]. This approach is based on the joint analysis of the stimulus and the response, including the measurement of three observable variables, namely the initial temperature of the skin T_0_, the apparent threshold AT, and the heating rate expressed as α. This paradigm allows one to determine the two key descriptors of a behavioral response to noxious heat in psychophysical terms, without using the reaction time t_R_ - the so-called ‘tail-flick latency’ (TFL). These descriptors are the behavioral threshold T_R_ and the behavioral latency L_R_, both of which are latent variables which are inaccessible with conventional methods. In addition we calculated the conduction velocities of the peripheral fibers that trigger the reaction and proposed an estimate of the central decisional latency Lδ - arguably the most interesting part of L_R_ to be investigated [7]. The usefulness of such an approach was demonstrated by providing new fundamental findings: the skin temperature, itself dependent on ambient temperature, very markedly influenced the behavioral threshold, the behavioral latency and the conduction velocity, but not the latency of the central decision-making process, at least in the range of ambient temperatures from 18 to 35°C. Finally, a simple model was proposed and verified experimentally, for computing variations of the TFL elicited by changes in either the power of the radiant heat source, the initial temperature of the skin or the site of stimulation on the tail.

First we will discuss successively the determined variables, namely threshold of the response (T_R_), conduction velocity of the fibers that triggered the reaction (V), central decisional latency (Lδ), latency of the response (L_R_) and reaction time (t_R_). Then we will consider the questions of the influence of central temperature and thermoregulation. We will conclude by questioning the construct validity of the tail-flick test and emphasize the importance of ambient temperature as the prevailing environmental factor during any behavioral testing.

### The true threshold of the behavioral reaction

We estimated 47.3°C as the grand-mean threshold from the tail of the mice. To the best of our knowledge, there are no available data for comparison of true or apparent behavioral thresholds in this species. The tendency of the distal parts of the tail to be more sensitive than the proximal, which was observed in the rat [1], did not reach the significance level here in the mouse, possibly because of the small length along which the stimuli could be applied. Overall, the threshold was higher than the corresponding 44.9°C in the rat. Interestingly, we determined the thresholds of the C-fiber evoked pain threshold from the human foot, hand and forehead with the same approach; these were: 42.4, 42.0 and 44.3°C respectively [14]. It is tempting to speculate that the sensitivity of the skin increases with the distance D, that the nociceptive signal must travel along the nerve. It is a matter of astonishment that the general properties of C-fibers, including the conduction velocity, are identical across species, whatever their place in phylogenies or their size [15]-[18]. It makes sense that a lower threshold compensates for the raised duration of the peripheral latency produced by extension of the peripheral path, thus maintaining an efficient protection of the more exposed parts of the body to injury, such as the extremities. It would be possible to verify the correctness of this hypothesis by further investigation in large-sized species.

A possible alternative (and/or complementary) explanation could be found in a weightier influence of stress in the mice. Stress responses are associated with sympathetically-mediated vasoconstrictions in the skin that lead to cooling of cutaneous territories, especially in the extremities. This was recently observed with infrared thermography in fear-conditioned rats, together with a 4.2°C increase of the nociceptive threshold to laser radiant heat stimulation [19], [20]. Increased reaction time to radiant heat was reported in mice following several unconditioned situations: elevated plus maze, exposure to predators, social isolation, defeat experience and predator odor (see refs in [21]). There is also a literature for the stress-induced hyperthermia paradigm used in anxiety studies. Increasing T_c_ was seen when removing mice from a group-housed cage to be placed individually in a restrainer, or by successive measurements of the rectal temperature (see refs in [22], [23]) Although the stress factor was minimized in our study because the mice were habituated to the container environment several times before the day of experiment and for one hour before the experiment, it cannot be excluded completely.

We observed that the behavioral threshold T_R_ rose as the surrounding skin temperature increased. This observation fits recent observations of a correlation between the temperature of the tail and the behavioral threshold for withdrawal in rats [1] and the increase in the C-fiber-evoked pain threshold produced by warming surrounding tissues in humans [14]. In all cases, a large area of warming was superimposed over a much smaller surface area for the application of test heat stimuli. This finding might be interpreted as resulting from a build-up process in the CNS resulting from population coding. Indeed, if one considers the peripheral information emanating from the tail, one sees a huge imbalance between information from the tiny site heated by the laser (<9 mm^2^) and the surrounding area (∼250 mm^2^). Such an imbalance is indisputably reflected in the firing of the corresponding populations of dorsal horn neurons, which means that the thermal picture of the tail received by the CNS will be more or less contrasted with the basal temperature. It is hypothesized that low background temperatures facilitate the detection of a nociceptive event - thus lowering the threshold - while higher background temperatures blur the detection of a nociceptive event - thus increasing the threshold. A dedicated study could confirm such a view and define the performance and limits of the bandwidth through a larger range of temperatures. Interestingly, noxious-evoked discharges of neurons in the lumbar spinal cord are inhibited by surrounding warming in both the cat and the rat [24], [25]. In addition, there is clinical evidence for the relief of pain by surrounding warming [26]-[28].

In any case, this factor of variation can be a further physiological source of variability of nociceptive responses when the surrounding ambient temperature gives rise to dynamic cyclic changes of the vasomotricity, as exemplified in Figure 2A.

### Conduction velocity of the fibers that triggered the reaction

The conduction velocities of the peripheral fibers that triggered the withdrawal were within the range of C-fibers. The temperature of the tail was the main determinant of this variable. The results were astonishingly similar regardless of the paradigm used (progressive heating ramps or brief heat shocks), as testified by the comparison of figures 6Bb and 7B. In both cases, the ratio of the conduction velocity at 30°C to the conduction velocity at 20°C was Q_10_ = 2.0, a value remarkably similar to the corresponding found in the rat by Benoist et al. [1] with the progressive heating ramps paradigm (Q_10_ = 2.1). The dependency of the conduction velocity of peripheral fibers on temperature is a classical electrophysiological notion [29], [30]. During recordings from C-fibers in anesthetized cats, Q_10_ values of 1.8 and 2.1 were reported from the saphenous and aortic nerves, respectively [31], [32]. These are very close to the values determined behaviorally in the present study. The tremendous impact of this factor can be envisaged by considering the 8°C-range of spontaneous variations of the temperature of the middle-tail which were observed when the room temperature exceeded 25°C (as illustrated in Figure 2).

Since the thresholds of activation of individual Aδ-fiber nociceptors are higher than those for C-fiber nociceptors [10]-[12], it was quite possible that we would have missed the activation of the former with the progressive heating ramps paradigm. This possibility was excluded by the second paradigm that involved high-power, short-duration, laser heat pulses. If the two types of fibers were involved in triggering the withdrawal responses, it could be assumed that the reactions recorded would be elicited by the fastest fibers, namely the Aδ-fibers. Since this was not the case, we concluded that Aδ-fibers did not participate in the triggering of the tail withdrawal in our experimental conditions. This observation is in keeping with earlier behavioral [1] and electrophysiological [33], [34] studies in the rat.

### Central decisional latency

We provided evidence for the homogeneity of V along the tail and across animals, for a given basal temperature of the skin. We introduced a correction for the increased temperature of the nerve in the core. The central decisional latency Lδ was thus estimated to be in the order of 87 ms in the progressive heating ramps paradigm. The corresponding values obtained with an identical approach in the rat was 132 ms. The reason for the ∼45 ms difference between species is unclear. The decisional latency, required for the central decision-making process, is initiated by the arrival (and/or the accumulation) in the CNS, of a sufficient amount of nociceptive information to order the triggering of the withdrawal. During this short period, modulation processes, notably those from supraspinal origin, have the opportunity to modify the withdrawal response. One can speculate that the modulation-producing systems are more efficient and/or powerful in the rat than in the mouse.

### Behavioral latency and reaction time

We observed that the behavioral latency was always less than 500 ms, even in situations that elicited the tardiest responses (stimulation of the extremity of the tail, cold environment). In situations that elicited the most rapid responses (stimulation of proximal tail, warm environment), this latency fell to ∼150 ms. The remaining part of the reaction time, t_R_, represents mainly the time for the heating process, Lϕ, and to a much lesser extent, the transduction time, Lτ, to achieve the threshold for the reaction, T_R_. Lτ can be inferred from isolated primary afferent neuron recordings: 35 ms as the time for half activation of the inward current elicited by pulses of noxious heat [35].

In any case, the sum t_R_ = (L_P_+L_R_) corresponds to the TFL measured in the conventional tail-flick test. In mice, the control TFL is generally restricted to the 2-3 seconds range, but can attain 5 or even 10 seconds (e.g. [36], [37]). If one considers a test - performed in a room at 25°C on the mid-tail - that would elicit a behavioral response within L_R_ ∼250 ms, one can conclude that 90, 95 or 97.5% of the measured variable is devoted to L_P_, a physical process time, when TFL = 2.5, 5 or 10 seconds, respectively. In other words, only 10, 5 or 2.5% of the TFL measures the targeted construct, i.e. a behavioral variable related to nociception. This part decreases when the TFL increases by lowering either the power of the radiant heat source or the basal temperature of the skin. Moreover, if the TFL is the only measured end-point elicited by a given source power, there is no way of knowing whether any variation was produced by changes of either T_0_ or T_R_ or both. In other words, using the reaction time of a behavioral response to an increasing heat stimulus as a “nociceptive index" must be challenged, as it does not achieve a good level of validity.

At this point, we wish to emphasize that both T_R_ and L_R_ were determined in the present paradigm without any use of t_R_, - each individual response being fully described by T_0_, AT and α. However, our results provided an opportunity to develop a predictive model of t_R_ which was fully verified following variations of the radiant heat source or the basal temperature of the skin.

### The central temperature

Endothermic mammals have the ability to maintain a constant core temperature T_c_ over a wide range of ambient temperatures T_a_. However, thermoregulation is challenging for small animals due to their large surface-area-to-mass ratio, which facilitates body heat loss. Many small endotherms must produce substantial amounts of endogenous heat to compensate for high heat loss during cold exposure. Moreover some of them such as mice are not permanently homeothermic, but during certain periods of the day enter a state of torpor [38], [39]. Torpor is characterized by a controlled reduction of T_c_, metabolic rate and other physiological functions - albeit not as deep as in hibernation - to conserve energy and this is associated with a fall in blood pressure and heart rate [40]. Daily torpor in mice is not restricted to starvation periods and includes the lowering of ambient temperatures [41], [42]. The possible occurrence of daily torpor in mice should be taken into consideration for comparison with rats of processes involving the CNS. Indeed, rats, which are roughly 10 fold heavier, never enter torpor [43]. In our experimental condition, a decline of core temperature was obvious from a 25°C ambient temperature onwards. At 18°C, the lowest ambient temperature tested, the mean core temperature was 34°C. Note that spontaneous torpor could produce lower hypothermia (<20°C). Since mice behave similarly to normo-thermic animals when T_c_ is above 26°C [44], it is probably because T_c_ never fell below 34°C in our short-lasting experiments (<90 min) that such a reduction did not affect the central decisional latencies. Clearly, our mice did not reach a torpor state, but they were unable to overcome body heat loss when they were submitted to the lower (<25°C) ambient temperatures, in spite of the vasoconstriction of the tail (see Figure 2B). In any case, one should be aware of such a potential factor of variation with mice taken from their litter to be introduced into an air-conditioned laboratory (typically ∼20°C). If they have the opportunity to select their environmental temperature, mice will choose an ambient temperature of approximately 30-32°C, which corresponds to the thermoneutral zone [44]-[46].

### Nociceptive reaction and thermoregulation

The main determinant of the TFL was the temperature of the tail, which itself was dependent on the interactions between the ambient temperature and the thermoregulatory processes, as pointed out already [1], [2], [47]-[51]. Note that the temperature of the tail governs two different effects: the time required to achieve a given temperature from the basal level and the behavioral threshold T_R_. It follows that a variation of the temperature of the tail elicits two opposite effects on the TFL. A drop in physical latency provoked by a skin temperature increase is compensated by a higher threshold, while a rise of the physical latency elicited by a skin temperature fall is countered by a decreased threshold. This can be the source of further difficulties in the interpretation of data, notably when the temperature variations are small and the intra-individual variability non-negligible. This was probably the case in experiments that supported the independence of the tail-flick reaction from cutaneous and central temperatures [52] (and see [53]).

Mogil and colleagues studied over several years, the genetics of pain by recording in apparently identical conditions and testing protocols, the reaction time following tail immersion in a 49°C water bath in a series of strains of mice [54]. This approach provides an opportunity to retrospectively identify and rank the sources of variability in an archival data set from a large body of independent observations [55], [56]. This analysis allowed the variance of this trait to be partitioned between environmental (42%), genetic (27%) and genetic × environmental (18%) sources. Such a result is not surprising, if one acknowledges the basal temperature of the tail as the main source of variance of the TFL.

### Summary and conclusions

In summary, we extent here to the mouse, the theoretical framework and the experimental paradigm based on random variations of the stimulus, originally described in the rat. We propose a psychophysical approach to a nociceptive behavior that enables the determination of four latent variables (T_R_, L_R_, Lδ, V_t_) that have been inaccessible with conventional methods. We believe that such an approach satisfies the repeated requests for improving nociceptive tests [2], [47], [49], [51], [57] and offers potentially heuristic progress for studying nociception on firmer physiological, behavioral and psychophysical grounds with the same approach in man and animals, this latter now including species of special interest like the mouse. We implore fellow scientists to renounce to use of TFL or any other reaction time of a response to an increasing stimulus as being a meaningful index of nociception. Although this paradigm appears to have some validity at first sight, it does not achieve the criterion of construct validity because it does not effectively measure the targeted construct, i.e. a quantitative nociceptive response, presumed to reflect an animal’s perception of pain [2]. When specifically considering the mouse which is a small species with a 30-32°C thermoneutral zone [44]-[46], [58], one should emphasize the need for very careful control of T_a_, as a prevailing environmental factor of variation, not only during housing [59] but also during any behavioral testing.

## Materials and Methods

### Ethic statement

Animal experiments were performed with permission of the Board of the Veterinarian Services of the French Ministry of Agriculture (permit number 75-151) in accordance with the National Institute of Health’s “Guide for the care and use of Laboratory animals", the European Communities Council Directive 86/609/EEC regulating animal research, and the ethics committee of the International Association for the Study of Pain [60], [61]. Procedures were approved by the Committee of Ethics for the Animal Experiment of our Institution (permit number Ce5/2011/037).

### Animals

Experiments were performed on males Swiss CD1 mice (Janvier, Le Genest Saint Isle, France; initial weights 25-30 g). They were acclimated for 1 week in groups of 4, in a room under a 12 h light-dark cycle, maintained at constant temperature (21±1°C), with a relative humidity of 50% and with free access to food and water. During the 5-7 days preceding the experiment, the mouse was placed 4 times for 1.5-3 hours, in the restrainer for habituation to the environment. The experiments were conducted between 9 am and 7 pm.

### General conditions for experimental protocol 1

We first evaluated the effects of ambient temperature on the temperature of the tail in 15 mice. Animals were introduced into a Plexiglas restrainer for two hours while the tail skin temperatures were recorded with the thermometric camera over six points on the tail. Such a procedure was replicated in each individual mouse at different ambient temperatures.

### General conditions for experimental protocols 2 and 3

The day before the experiment, the entire tail was depilated with a depilatory cream (Hair removal cream Dermo-Tolerance, Vichy® Laboratories, Cusset, France.) while the animals were positioned in the Plexiglas restrainer. The cream was applied for 10 min, followed by thorough rinsing. The animal was habituated to this environment for one hour before the experiment. A colonic thermocouple was inserted 2.5 cm beyond the anal sphincter and fixed with adhesive tape [58]. During the course of testing (see below), stimulation was never applied during any behavioral or postural adjustment of the animal. No signs of burning were seen the day following testing. At the end of the experiments, the animal was sacrificed using an overdose of pentobarbital, and autopsied. The L3 vertebra was identified and the distances (D) between the stimulation sites and this vertebra were measured. This level was considered as the main entry zone in the cord for afferent signals from the tail by analogy with the rat (see refs in [1]).

The second series of experiments was performed in eight mice to assess the nociceptive reaction elicited by stimulation with variable laser energy (70-350 mJ), delivered in a pseudo-random order until tail withdrawal. Three points of stimulation, 25 mm apart, were stimulated on each side of the tail. The stimuli were applied successively to these sites, the left and the right side being stimulated alternately. The experiments were reiterated over 3 sessions with cold (17-22°C), medium (24-27°C) and hot (32-35°C) ambient temperatures. A minimum of five minutes was allowed between the applications of stimuli to a given site.

In the third series of experiments (5 mice), high-power, short-duration laser heat pulses (30 ms; 45-75 mJ according to the basal temperature of the tail T_0_) were applied to various sites on both sides of the tail, 10 mm apart, from the proximal to the distal regions. Shifting all sites by 5 mm, so that a given site was stimulated once, allowed the reiteration of the series. The procedure was applied twice during a session. The experiments were reiterated in five mice over three sessions at three different ambient temperatures.

### The stimulus

We used a laser stimulator (CO_2_LSD, SIFEC, Ferrière, Belgium) for the following reasons [62]: (1) it is an infrared monochromatic radiant source with a long wavelength (10.6 μm) for which the absorbance is almost total whatever the pigmentation of the skin and the incidence of the beam; (2) the transparency of skin is weak (∼100 μm), so that the calorific energy absorbed at the level of the cutaneous surface propagates towards nerve endings sensitive to thermal variations, which are localized above the dermo-epidermic junction (60-120 μm depth); (3) the temporal and spatial profile of the calorific energy is well determined; (4) given the high power density of the laser, it is possible to apply abrupt heating. The surface area for stimulation was a circle determined by the Gaussian power profile of the laser beam. We chose a diameter of 3.4 mm, for which lateral diffusion of heat by conduction was negligible for at least 2.5 seconds (the longest reaction time recorded in the present study), as checked by the linearity of the functions ΔT^2^ = f(t). Beyond this period, diffusion processes could gradually and significantly thwart the temperature increase.

During laser stimulation, the temperature increases proportionally with the square root of time according to the law of radiant heat transfer T = T_0_+a_*_t^0.5^ (see Figure 3 left) or, expressed in terms of temperature variation: ΔT = T – T_0_ = a_*_t^0.5^ [8]. This quadratic relationship becomes linear in t by squaring the two terms of the equation: ΔT^2^ = α_*_t, with α = a^2^ (slope of the straight lines in the right graphs of Figure 3). The constant term a is proportional to the power density (Q) of the laser, according to the relation a = K_*_Q = K_*_q/S, where K is a composite constant grouping together the biophysical properties of the skin, S the stimulation surface area (mm^2^) and q the laser power (mW). The linearity of this relationship was verified independently with a radiometer (13PEM001, Melles-Griot, The Netherlands) and fitted with a linear least squares regression procedure yielding a = 4.17_*_10^-3^_*_q with zero intercept and R = 0.933. It follows that α is proportional to q^2^. To reach a satisfactory level of reproducibility, the laser beam must be perpendicular to the stimulated surface because the angle of incidence influences power density. However, the skin is never flat and the tail is a conical cylinder. In order to minimize these sources of variability, the beam axis was targeted perpendicular to the axis of the tail. The beam was adjusted to 45° with respect to the vertical, in order to elicit a contralateral withdrawal movement. Doing this allowed stimulation of the right and left side of the tail at a given rostro-caudal level.

In the second series of experiments, the power of stimulation was chosen at random in a uniform distribution ranging from 100-350 mW in order to provoke responses within less than 2.5 s without damaging the skin. In these conditions, the slope α was in the 0.07-3.2°C^2^/ms range and the maximum temperature reached at the actual moment of the reaction was always lower than 70°C. The laser beam duration was maximal of 2.5 s or discontinued by the experimenter as soon as the tail moved.

### The thermometric camera

A JADE MWIR (3-5 μm optical bandpass) camera (CEDIP Infrared Systems, Croissy-Beaubourg, France) with a 500 ms integration time, which supplied images of 320×240 pixels at 100 Hz with a sensitivity of 0.02°C at 25°C was used. The camera was placed upright to the zone of stimulation and was controlled by the software Cirrus (CEDIP Infrared Systems, Croissy-Beaubourg, France). It was calibrated by means of a black body (CI SR80 CI Systems, Migdal Haemek, Israel). The software Altair (CEDIP Infrared Systems, Croissy-Beaubourg, France) allowed the monitoring of the spatial and temporal evolution of the temperature at the level of the stimulated surface area with 0.3 mm and 5.8 ms resolutions, respectively. The recording was triggered 350 ms before the application of the stimulus.

### Measuring the temperature of the skin

The measurement of temperature at the skin surface is justified by convenience of use, its non-invasive character and the possibility of extrapolating the underlying subcutaneous temperatures by modeling. This temperature represents only an approximation of the temperature reached at the level of the nociceptors, which are located at the dermo-epidermal junction, at an average depth of 100 μm [8], [63], [64]. The temperature reached by the various layers within the skin can be estimated by modeling and simulation [63]-[66]. We verified previously that the temperature reached at the dermo-epidermal junction in our experiments was slightly lower, but indeed close (<1.5°C) to the measured surface temperature (see Figure 10 in Benoist et al [1]).

Although we filmed the full surface area of stimulation, we chose to investigate further the temporal evolution of the warmest pixel. From the physical standpoint, this choice was justified by the Gaussian profile of the beam, reflected by an equivalent spatial profile of the temperature increment). Knowing the highest value and the diameter of the temperature profile allows anyone to reconstruct the whole picture.

The ambient temperature (T_a_) was measured on a piece of wood close to the tail of the animal. In some few cases (abrupt vasodilatation, piece of wood too much near the tail), the piece of wood could be slightly influenced by the temperature changes of the tail - by emission, conduction and/or convection processes -. Such artifacts (<0.5°C) were deleted (e.g. Figure 2A, near 50 min point).

### Analysis of thermographic films

The analysis of the thermographic films involved the following steps: (1) determination of the zone of interest in the recorded scene (i.e. heating spot at the end of stimulation); (2) determination of the initial mean temperature T_0_ in this zone; (3) calculation of the temporal evolution of the warmest pixel in this zone. This pixel corresponded to the top of the Gauss curve, which characterizes the spatial profile of the thermal rise and the highest temperature is referred as T_max_; (4) determination of the moment when the tail withdrawal occurs, i.e. determination of the reaction time t_R_. This reaction time is measured by counting the picture number of the film between the stimulus onset and the withdrawal time, which is then converted into time by considering the frequency of the images (100 Hz, i.e. a frame each 10 ms).

In summary, the analysis of an individual temperature curve included the following steps: (1) transforming the temperature difference with regard to the initial temperature ΔT = f(t); (2) raising to the square ΔT^2^ = f(t); (3) checking the linearity of this curve; and (4) determining the value of the slope α of this curve. Each behavioral trial was summarized finally by four accessible quantities measured independently, namely the initial skin temperature T_0_, the maximum temperature T_max_, the slope α and the reaction time t_R_.

### Determination of the threshold and the latency of the reaction

On completion of the analysis of a series of behavioral trials elicited from a given skin territory, the stability of the basal temperature T_0_ was checked by considering the histograms of distribution and excluding trials for which T_0_ deviated from the mean with more than two standard deviations. Since T_0_ remained stable during the experimental procedures, we could infer the threshold T_R_ and the latency L_R_ of the reaction - presumably constant -, from a series of trials where the power of the radiant heat source varies to produce an appropriate range of α. We used two approaches - graphical and mathematical.

One can modify the representation by adjusting the origin of the time scale of each individual curve for heating to the actual moment of the reaction: t – t_R_ (See Figure 3B and 4A). Such a change of origin allows one to visualize the back timing of events and to identify the point –L_R_ on the abscissa and T_R_ on the ordinate. Because of the stochastic nature of the psychophysical responses, the points of intersection are distributed in the time vs. temperature plane (white circles in Figure 3B and 4A).

Considering squared temperature variations ΔT^2^ yields the following expressions: ΔT_R_^2^ = α_*_(t_R_ – L_R_) [**equation 1**] and ΔAT^2^ = α_*_t_R_ [**equation 2**] (Figure 1A). One can then substitute α.t_R_ of equation 2 in equation 1 to obtain after rearrangement a linear relationship ΔAT^2^ = f(α): ΔAT^2^ = ΔT_R_^2^+L_R*_α [**equation 3**]. By plotting ΔAT^2^ as a function of α, the points aligned themselves quite well along a straight line (Figure 1B and see Figure 3B), the slope and intercept of which correspond to L_R_ and ΔT_R_^2^, respectively. Evaluation of the latency L_R_ and of the threshold T_R_ = T_0_+√ΔT_R_^2^ by this method is justified as α is measured independently of ΔAT^2^. In practice, the global analysis of the individual curves from a series of tests included the following steps: (1) building the initial temperature T_0_ histogram; (2) exclusion of data for which T_0_ deviated from the mean by more than 1°C; (3) construction of the graph ΔAT^2^ = f(α); (4) checking the linearity of the function ΔAT^2^ = f(α); (5) Determination of T_R_ and L_R_.

### Calculation of the conduction velocity of the fibers that trigger the reaction

Conduction velocities V_t_ of the fibers of the coccygeal nerve traveling within the tail of the animal and which triggered the reaction, were calculated on the basis of the linear relationship between the distance D from the stimulation site to the dorsal horn entry zone and the corresponding calculated psychophysical latency, L_R_ = f(D) = y+D/V_t_, where y is the intercept with the ordinate and 1/V the slope of the straight line (See Figure 4D).

The dependency of the conduction velocity of peripheral fibers on temperature is a classical electrophysiological notion [29]-[31]. We have previously verified in the rat [1], that the slope of the regression lines of the relationship t_R_ = f(D) increased when the tail temperature decreased. In other words, the warmer the tail was, the faster the conduction velocity was, with a highly significant linear relationship Vt = f(T_0_) = a_*_T_0_+b, between the temperature of the tail and the conduction velocity (with a and b as constant terms). Such a relationship was verified here (see results). Knowing the core temperature of the mouse provides the possibility of calculating the conduction velocity of fibers at core temperature in the trunk (T_c_): V_c_ = V_t_+a_*_(T_c_ – T_0_).

### Determination of the tail-trunk interface

The V_t_ to V_c_ change occurs at the tail-core interface where the temperature of the nerves increases from T_0_ to the core temperature T_c_. This interface can be estimated statistically in the D-L_R_ plot by considering the overall cluster of the intersection points of each straight line with the others (see Figure 6Ba). A crossed tabulation of the slope and intercept of each straight line was used to compute the median values of the coordinates of these intersection points, which are estimations of D_c_ = length of the coccygeal nerve traveling within the trunk at core temperature and (Lπ_c_+Lδ+Lμ), respectively.

### Estimation of the decisional latency

Knowing: (1) the distance D_c_ corresponding to the interval between the dorsal horn entry zone and the tail-trunk interface, where the conduction velocity increases from V_t_ to V_c_; (2) the core temperature T_c_; and (3) the conduction velocity at core temperature V_c_ in each individual case, an estimation of the decisional latency Lδ can be inferred from the intercept y_t_ of the regression line L_R_ = f(D) with the ordinate (Figure 1C).

The temperature increases over the length (D_c_) of the coccygeal nerve traveling within the core of the animal, which is controlled by thermoregulation processes. Therefore, one should dissociate the peripheral latency (Lπ) into two components, successively related to the tail (Lπ_t_) and the core (Lπ_c_), with two different conduction velocities (V_t_<V_c_): Lπ = Lπ_t_+Lπ_c_ = (D – D_c_)/V_t_+D_c_/V_c_. One can deduce the virtual regression line L_R_ = f(D) within the core, from the actual line measured from the part of the coccygeal nerve traveling within the tail of the animal.

The slope (1/V) of the virtual regression line L_R_ = f(D) is reduced in the part of the nerve traveling within the core of the animal. The intercept y_c_ of the virtual regression line L_R_ = f(D) is increased by as much: y_c_ = y_t_+D_c*_(1/V_t_ – 1/V_c_). This intercept corresponds to the part of the latency of the reaction that is not devoted to the progress of nociceptive signals along afferent fibers. According to Luce [7], the period following the arrival of signals in the CNS corresponds sequentially to the central decisional latency Lδ and the motor latency Lμ, time from motoneuron activation up to the shortening of the muscle, i.e. y_c_ = Lδ+Lμ. Lμ is unknown in the mouse but has been estimated as 4 ms in the rat [9]. One can easily imagine that the motor latency Lμ is still shorter in the mouse, and negligible as compared to the decisional latency. From these considerations, the conclusion is reached that our approach provides sufficient information to estimate the central decisional latency Lδ, the most interesting part of t_R_ to be investigated [7]: Lδ = y_c_ – Lμ = y_t_+D_c*_(1/V_t_ – 1/V_c_) – Lμ.

### High power short laser pulses

High power (T**_max_** ∼70°C) but very short (30 ms) laser pulses of heat (see insert in Figure 7A) were applied to various sites along the tail and the corresponding reaction times measured. The conduction velocities of the fibers in the part of the coccygeal nerve traveling within the tail of the animal were calculated conventionally.

### Statistical analyses

Least squares linear regressions and one-way analyses of variance (ANOVA) were used for statistical purposes. Calculations were performed with the statistical software Statview® 5.0 and Statgraphics® Plus 5. Other calculations were made with the software Mathcad®. Results were considered significant at P<0.05. Data are expressed as means (± confident interval 95%).
